# *Neoplecostomus doceensis*: a new loricariid species (Teleostei, Siluriformes) from the rio Doce basin and comments about its putative origin

**DOI:** 10.3897/zookeys.440.8203

**Published:** 2014-09-15

**Authors:** Fábio F. Roxo, Gabriel S. C. Silva, Cláudio H. Zawadzki, Claudio Oliveira

**Affiliations:** 1 Universidade Estadual Paulista, UNESP, Departamento de Morfologia, Laboratório de Biologia e Genética de Peixes, 18618-000, Botucatu,São Paulo State, Brazil; 2 Universidade Estadual de Maringá, Departamento de Biologia, Núcleo de Pesquisas em Limnologia, Ictiologia e Aquicultura (Nupélia), 87020-900, Maringá, Paraná State, Brazil

**Keywords:** Brazilian shield, catfishes, freshwater, ichthyology, Neoplecostominae, Neotropical fishes, Ostariophysi

## Abstract

A new species of *Neoplecostomus* is described from the rio Doce basin representing the first species of this genus in the basin. The new species is distinguished from its congeners by having enlarged, fleshy folds between dentaries, two or three series of developed papillae anterior to premaxillary teeth and a adipose-fin membrane present, and by lacking enlarged odontodes along snout lateral margins in mature males, a well-developed dorsal-fin spinelet wider than dorsal-fin spine base, lower number of lateral-line figs and developed membrane on the dorsal portion of the first, second and third pelvic-fin branched rays. Additionally, we present a brief discussion of biogeographic scenarios that may explain the distribution of the new species in the rio Doce basin. We suggested that the ancestral lineage of the new species reached the rio Doce from the upper portions of rio Paraná drainages about 3.5 Mya (95% HPD: 1.6–5.5) indicating a colonization route of the *N. doceensis* ancestral lineage from the south end of Serra do Espinhaço, probably as a result of headwater capture processes between the upper rio Paraná and rio Doce basins.

## Introduction

Neoplecostominae currently includes six genera: *Neoplecostomus*, *Isbrueckerichthys*, *Kronichthys*, *Pareiorhaphis*, *Pareiorhina* and *Pseudotocinclus* (Armbruster 2004; Chiachio et al. 2008; Roxo et al. 2012a, b) and more than 50 valid species (Eschmeyer and Fong 2014) distributed throughout the southeastern drainage regions in South America, from Rio Grande do Sul to Bahia states, except for *Pareiorhaphis regani*, which occurs in the rio Negro, in the Amazon basin.

Since Langeani (1990), the genus *Neoplecostomus* has been diagnosed as having a conspicuous series of enlarged papillae just posterior to the dentary teeth, which are larger than those on the remaining portions of the lower lip, the abdomen covered with figlets forming either a pentagonal or hexagonal shield, and the canal bearing fig on the cheek and the dorsal locking mechanism absent. Presently, the genus includes 13 species (Eschmeyer 2014): *Neoplecostomus paranensis* Langeani, 1990, *Neoplecostomus corumba* Zawadzki, Pavanelli & Langeani, 2008, *Neoplecostomus selenae* Zawadzki, Pavanelli & Langeani, 2008, *Neoplecostomus yapo* Zawadzki, Pavanelli & Langeani, 2008, *Neoplecostomus botucatu* Roxo, Oliveira & Zawadzki, 2012, *Neoplecostomus bandeirante* Roxo, Oliveira & Zawadzki, 2012, *Neoplecostomus langeanii* Roxo, Oliveira & Zawadzki, 2012, all from the upper rio Paraná basin; *Neoplecostomus franciscoensis*, Langeani, (1990) from the rio São Francisco basin; *Neoplecostomus microps* (Steindachner, 1877), *Neoplecostomus variipictus* Bizerril, 1995, and *Neoplecostomus granosus* (Cuvier & Valenciennes, 1840) from the rio Paraíba do Sul basin; *Neoplecostomus espiritosantensis* Langeani, 1990 from rio Jacu basin and *Neoplecostomus ribeirensis* Langeani (1990) from rio Ribeira de Iguape basin.

An examination of the fish collections at the LBP (Laboratório de Biologia e Genética de Peixes de Botucatu – São Paulo); MCNIP (Museu de Ciências Naturais da PUC Minas – Minas Gerais); MZUSP (Museu de Zoologia de São Paulo – São Paulo); and NUP (Coleção Ictiológica do Núcleo de Pesquisas em Limnologia, Ictiologia e Aquicultura, Universidade Estadual de Maringá – Paraná) revealed the existence of an undescribed species of *Neoplecostomus* from the rio Doce, the first species of the genus described from this basin. Additionally, we present a brief discussion of biogeographic scenarios that may explain the distribution of the new species in the rio Doce basin.

## Material and methods

Measurements and counts were taken on the left side of the specimens. Body fig nomenclature follows Schaefer (1997) and measurements follow Langeani (1990), modified by Zawadzki et al. (2008), and are shown in Table 1. All measurements were taken point to point with digital callipers to the nearest 0.1 mm. Specimens were cleared and stained (c&s) according to the method of Taylor and Van Dyke (1985). Dorsal-fin ray counts included the spinelet as the first unbranched ray. Vouchers were deposited in the collections of the (LBP) Laboratório de Biologia e Genética de Peixes, Universidade Estadual Paulista, Botucatu, Brazil; (MCNIP) Museu de Ciências Naturais da PUC Minas, Minas Gerais, Brazil; (MZUSP) Museu de Zoologia da Universidade de São Paulo, São Paulo, Brazil; (NUP) Coleção Ictiológica do Nupélia, Universidade Estadual de Maringá, Maringá, Brazil. The scientific names of the species follow the International Code of Zoological Nomenclature (International Commission on Zoological Nomenclature 1999).

**Table 1.** Morphometric and meristics of *Neoplecostomus doceensis* (holotype and paratypes). SD = standard deviation.

	*Neoplecostomus doceensis* n = 26				
	Holotype	Min	Max	Mean	SD
SL	101.1	40.9	101.1	72.4	16.3
**Percents of SL**					
Predorsal length	43.2	42.5	47.0	43.8	0.9
Head length	31.1	31.0	33.1	32.3	0.6
Head width	28.2	25.7	28.5	27.5	0.7
Cleithral width	26.3	25.8	28.7	27.1	0.8
Occipital-dorsal distance	12.1	10.7	13.8	12.3	0.7
Thoracic length	17.9	14.6	18.6	17.2	1.1
Interdorsal length	19.9	16.9	22.0	18.9	1.3
Caudal peduncle length	28.3	27.3	38.7	31.3	3.2
Caudal peduncle depth	8.7	6.5	8.7	7.4	0.5
Body depth	19.6	15.3	19.6	17.5	1.2
Preanal length	65.1	59.0	67.0	64.3	1.9
**Percents of HL**					
Head width	90.7	79.8	90.8	85.2	2.6
Head depth	56.5	47.1	57.1	51.9	2.7
Snout length	69.1	62.7	69.2	65.9	1.9
Orbital diameter	7.9	7.0	11.2	8.8	1.2
Interorbital width	32.5	29.3	34.1	31.6	1.3
Mandibullary width	18.8	12.5	22.4	18.2	2.6
**Other percents**					
Snout length/Orbital diameter	11.4	10.6	17.7	13.3	1.9
Interorbital/Orbital diameter	24.3	23.3	33.7	27.8	3.4
Interorbital/mandibullary width	57.9	44.3	74.4	58.6	8.4
Predorsal length/first ds ray length	46.0	41.8	51.1	46.1	1.8
Caudal peduncle length/Caudal peduncle depth	30.6	18.5	30.7	23.7	3.0
Pelvic-fin length/Caudal peduncle depth	33.9	25.4	36.5	29.5	2.6
Lower cd spine/Caudal peduncle depth	31.6	22.3	35.4	26.7	2.8
**Meristics**	**Holotype**	**Min**	**Max**	**Mode**	**SD**
Lateral-line figs	27	25	29	27	1
Predorsal figs	6	4	7	6	1
Plates of dorsal-fin base	6	5	6	6	0
Plates between dorsal and caudal	15	15	18	16	1
Plates between adipose and caudal	5	5	6	5	0
Plates between an and caudal	11	10	13	12	1
Premaxillary teeth	26	14	33	22	6
Dentary teeth	20	12	35	20	6

## Results

### Neoplecostomus doceensis sp. n.

Taxon classificationAnimaliaSiluriformesLoricariidae

http://zoobank.org/28057609-4191-4808-B6C0-7DABFD0E73D2

Figure 1 ; Table 1

Neoplecostomus sp. 9 – Roxo et al. 2012a: 2443 [phylogenetic relationships]. – Roxo et al. 2012b: 38 [phylogenetic relationships].

#### Holotype.

MZUSP 115486 (1 male 101.1 mm SL), Brazil, Minas Gerais State, municipality of Ouro Preto, córrego Bananeiras, affluent of rio Gualaxo do Norte, rio Doce basin, 20°14'20"S, 43°28'40"W, Abril 2010, BP Maia.

#### Paratypes.

All from Brazil, Minas Gerais State, rio Doce basin (97 specimens).

LBP 1098 (1 female 40.9, 1 male c&s 57.3 mm SL), municipality of Alto Rio Doce, rio Xopotó, 21°08'56"S, 43°23'58"W, October 2001, JC Oliveira, Al Alves, LR Sato. LBP 12261 (3 females 28.7–46.2 mm SL), municipality of Desterro do Melo, rio Xopotó, 21°09'09"S, 43°31'37"W, October 2011, A Ferreira, FF Roxo, GSC Silva. LBP 18981 (2 females 58.3–82.0 mm SL), uncertain location of the rio Piranga, 19 November 2000, JC Oliveira, OT Oyakawa. MCNIP 439 (3 males 80.6–86.7 mm SL), municipality of São José do Mantimento, rio José Pedro, affluent of rio Manhuaçu, 20°04'45"S, 41°44'00"W, 27 February 2012, TC Pessali, TA Barroso. MCNIP 1169 (1 female 59.4 mm SL, 4 males 75.0–100.3 mm SL), municipality of São José do Mantimento, rio José Pedro, affluent of rio Manhuaçu, 20°00'57"S, 41°44'07"W, 25 September 2013, TC Pessali, GM Santos. MZUSP 69368 (2 females 70.6–88.0 mm SL), municipality of Coroací, rio Suaçuí Pequeno (at bridge of Procópio), 18°41'38"S, 42°12'50"W, 29 April 2001, AM Zanatta. MZUSP 80971 (3 males 70.8–96.3 mm SL), municipality of São Luiz, rio Manhuaçu, 20°20'11"S, 42°042'48"W, 21 April 2002, CBM Alves. MZUSP 94487 (1 female 54.1 mm SL), municipality of Alto Rio Doce, rio Xopotó, rio Doce basin, 21°04'04"S, 43°27'50"W, 11 July 2007, OT Oyakawa. MUZSP 94505 (6 females 31.4–40.5 mm SL) municipality of Desterro do Melo, rio Xopotó, rio Doce basin, 21°08'53"S, 43°30'46"W, 10 July 2007, OT Oyakawa. MUZSP 94514 (1 female 35.8 mm SL) municipality of Alto Rio Doce, rio Xopotó, rio Doce basin, 21°03'11"S, 43°26'46"W, 10 July 2007, OT Oyakawa. MUZSP 94527 (7 females 33.7–41.5 mm SL) municipality of Desterro do Melo, rio Xopotó, rio Doce basin, 21°09'10"S, 43°31'49"W, 10 July 2007, OT Oyakawa. MZUSP 94542 (1 male 53.1 mm SL, 7 females 37.7–53.9 mm SL) municipality of Desterro do Melo, rio Xopotó, rio Doce basin, 21°09'10"S, 43°31'28"W, 10 July 2007, OT Oyakawa. MZUSP 107368 (2 males 61.1–84.5 mm SL, 3 females 47.8–79.5 mm SL), uncertain location of the rio Piranga, 19 November 2000, JC Oliveira, OT Oyakawa. MZUSP 109327 (9 males 55.3-90.6 mm SL, 29 females 32.2–93.6 mm SL), municipality of Manhuaçu, affluent of the rio Manhuaçu, 20°17'34"S, 42°03'41"W, October 2008, TC Pessali. MZUSP 109339 (1 male 51.7 mm SL, 2 females 53.8–69.6 mm SL) collected with holotype. MUZUSP 110931 (2 males 63.7–80.7 mm SL), municipality of Mariana, rio Gualaxo do Sul, 20°30'17"S, 43°24'40"W, July 2012, LF Salvador, LAC Missiaggia. NUP 17003, (1 female 83.2 mm SL, 2 males 96.6–100.3 mm SL), municipality of São José do Mantimento, rio José Pedro, affluent of rio Manhuaçu, 20°00'57"S, 41°44'07"W, 25 September 2013, TC Pessali, GM Santos. NUP 17004, (3 males 89.4–97.7 mm SL), municipality of São José do Mantimento, rio José Pedro, affluent of rio Manhuaçu, 20°04'45"S, 41°44'00"W, 27 February 2012, TC Pessali, TA Barroso.

**Figure 1.**
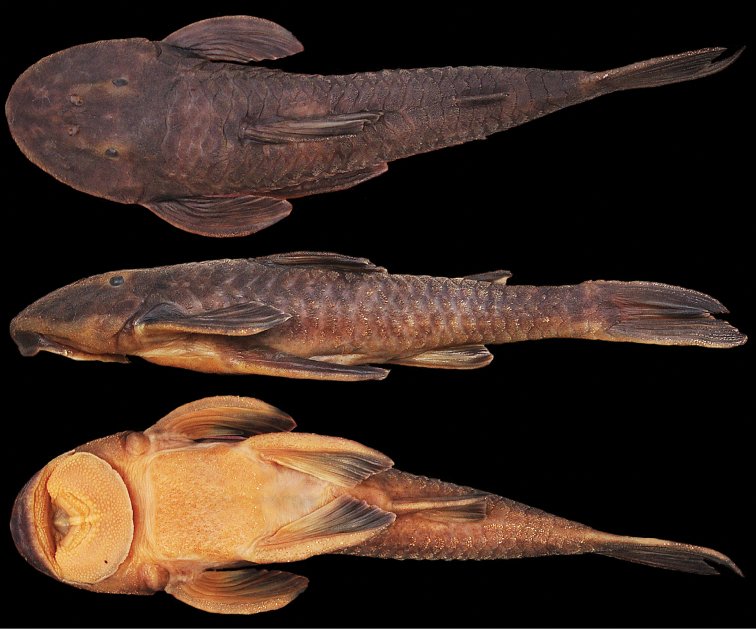
*Neoplecostomus doceensis*, MZUSP 115486, male, 101.1 mm SL, holotype from the affluent of rio Gualaxo do Norte, rio Doce basin, municipality of Ouro Preto, Minas Gerais State, Brazil.

#### Non-type specimens.

LBP 1096 (2 unsexed 54.4–57.7 mm SL), municipality of Alto Rio Doce, rio Xopotó, 21°08'56"S, 43°23'58"W, October 2001, JC Oliveira, AL Alves, LR Sato.

#### Diagnosis.

**Figure 2.**
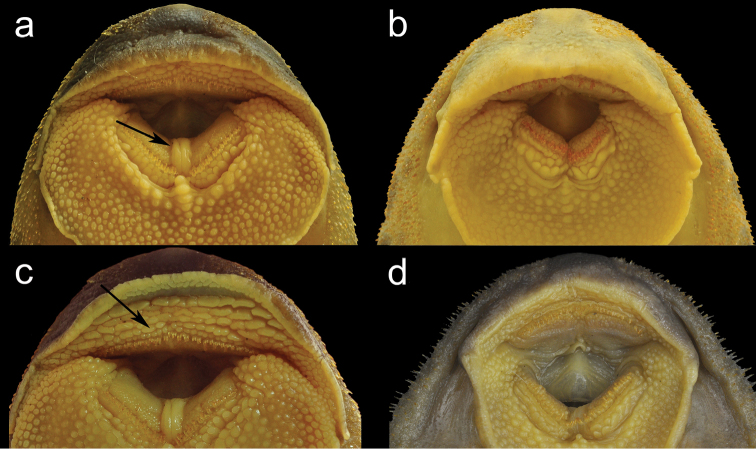
**a** *Neoplecostomus doceensis*, MZUSP 115486, 101.1 mm SL, male, holotype, arrow showing the developed papillae between dentaries **b** *Neoplecostomus botucatu*, MZUSP 110364, 98.6 mm SL, male, paratype, lacking the papillae between dentaries **c** *Neoplecostomus doceensis*, MZUSP 115486, 101.1 mm SL (male), holotype, arrow showing the developed papillae series anterior to premaxillary teeth **d** *Neoplecostomus selenae*, MZUSP 51889, 101.7 mm SL, male, paratype, lacking the papillae series anterior to premaxillary teeth.

*Neoplecostomus doceensis* is distinguished from all other congeners by having enlarged, fleshy folds between dentaries in all specimens, more evident in mature males, Fig. 2a (vs. absence of the enlarged fleshy folds, Fig. 2b). The new species can also be distinguished from all congeners by the presence of two or three series of well-developed papillae anterior to premaxillary teeth, Fig. 2c (vs. papillae poorly developed or absent Fig. 2d). Additionally, the new species can be distinguished from *Neoplecostomus botucatu* and *Neoplecostomus paranensis* by the presence of a fully-developed adipose fin (vs. lacking or reduced adipose fin); from *Neoplecostomus selenae* by moderately-sized odontodes along lateral margins of snout and snout without swollen skin in mature males (vs. presence of large-sized odontodes surrounded by swollen skin along lateral margins of snout in mature males); from *Neoplecostomus franciscoensis* and *Neoplecostomus ribeirensis* by having a well-developed dorsal-fin spinelet, wider than dorsal-fin spine base (vs. absent or narrower than dorsal-fin spine base); from *Neoplecostomus microps* and *Neoplecostomus variipictus* by a higher number of dentary teeth 12–35 (vs. 5–12 and 7 respectively); from *Neoplecostomus granosus* by having a lower number of lateral-line figs, 25–29 (vs. 34–43); from *Neoplecostomus langeanii* by the presence of a developed membrane on the dorsal portion of the first, second and third pelvic-fin branched rays (vs. lacking).

#### Description.

Counts and measurements are presented in Table 1. Body robust, elongated and depressed, greatest width at cleithrum (25.8–28.7% SL), narrowing to caudal peduncle. Dorsal profile of the head elevating and gently convex from snout tip to posterior margin of nares, straight to slightly concave to posterior margin of parieto supraoccipital, straight to dorsal-fin origin. Dorsal profile of trunk slightly concave and descending from dorsal-fin origin to adipose-fin origin, almost straight and descending to first procurrent caudal-fin ray; greatest body depth at dorsal-fin origin (15.3–19.6% SL). Ventral profile slightly convex from snout tip to anal-fin origin; concave at anal-fin region, straight and ascending to lower caudal-fin ray. Trunk and caudal peduncle almost ellipsoid in cross-section, rounded laterally and almost flat dorsally and ventrally.

Dorsal body surface completely covered by dermal figs, except for a naked area around dorsal-fin base and a small naked area at snout tip. Ventral head surface naked except for a fig bearing odontodes in front of gill openings. Abdomen with conspicuous, small dermal figlets between insertions of pectoral and pelvic fins, forming a thoracic shield surrounded by naked areas. Abdominal figlets densely covered by backward-oriented odontodes, their tips round. Head wide (79.8–90.8% HL) and depressed (47.1–57.1% HL). Head and snout rounded in dorsal view; interorbital space straight to slightly concave in frontal view.

Snout tip with a weak ridge between nares, sometimes absent, more evident in larger specimens. A weak ridge from middle of snout to superior margin of orbit. Moderate-sized odontodes along lateral margins of snout, more evident in mature males. Eye moderately small (7.0–11.2% HL) and dorso-laterally placed; lips well developed and rounded; lower lip almost reaching pectoral girdle and covered with papillae, wider anteriorly. Enlarged fleshy folds among dentary, more evident in mature males (Fig. 2a). Two to three irregular and conspicuous rows of large and transversally flattened papillae along and posterior to dentary teeth and anterior to premaxillary teeth (Fig. 2c). Maxillary barbel very short, coalesced, usually its tip not free from lower lip. Teeth long, slender and bicuspid; mesial cusp longer than lateral; dentary ramus forming an angle of approximately 125–130°.

Dorsal fin II,7; origin slightly posterior to pelvic-fin origin; dorsal-fin spinelet semicircular and wider than dorsal-fin spine base (spinelet hardly visible in some specimens, but always present); dorsal-fin locking mechanism not functional; dorsal-fin posterior margin straight to slightly rounded, reaching end of pelvic-fin rays when adpressed. Adipose-fin well developed and always present, preceded by azygous fig. Pectoral-fin I,6; unbranched ray depressed and curved inward (more pronounced in larger specimens), shorter than longest branched ray; posterior margin slightly concave, almost reaching half pelvic-fin ray length when adpressed; unbranched ray anteroventrally covered with backward-oriented odontodes. Pelvic-fin I,5; posterior margin nearly straight, reaching anal-fin insertion when adpressed; pelvic-fin unbranched ray ventrally flattened, with dermal flap on its dorsal surface in males; first and second branched rays also with dermal flap on its dorsal surface in males; unbranched ray anteroventrally covered with backward-oriented odontodes. Anal-fin I,5; posterior margin nearly straight; unbranched ray ventrally covered with back-oriented odontodes. Caudal-fin I,7,7,I; bifurcated; lower spine longer than upper; pectoral spine and unbranched pelvic-fin rays with odontodes on lateral and ventral portions.

#### Color in alcohol.

Ground color of dorsal surface of head and body dark brown to lighter brown in some specimens. Head with a pale spot on naked area of snout tip; orbital margin slightly lighter, mainly on its superior portion; small pale spot on interorbital space; lateral margin of snout usually lighter than rest of dorsal surface of head. Body dorsal color pattern in most specimens with four transverse light bands: first through supraoccipital, second in middle of dorsal-fin, third posterior to dorsal-fin, fourth posterior to adipose-fin. Body lateral portion with an upper darker and a lower lighter, just below lateral line. Dorsal, pectoral, pelvic, anal and caudal fins with hard visible irregular series of dark spots on rays. Ventral surface of head and body light brown.

#### Sexual dimorphism.

Males with papilla at the urogenital opening and a membrane along the dorsal portion of the unbranched pelvic-fin ray. Males seem to reach a greater length.

#### Distribution.

**Figure 3.**
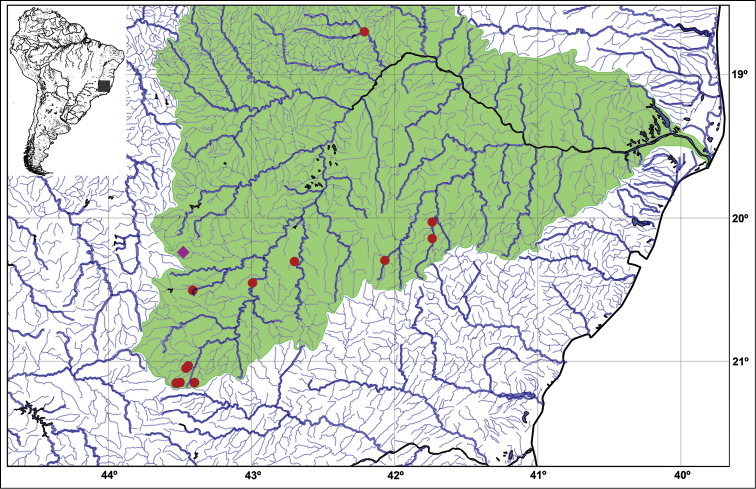
Map showing the type locality (pink diamond), 20°14'20"S, 43°28'40"W, and paratypes (red circles) of *Neoplecostomus doceensis* at the rio Doce basin (green highlighted drainages). See Distribution and Paratypes sections for details about each paratype localities.

*Neoplecostomus doceensis* is known from thirteen localities: one at rio Gualaxo do Norte, one at rio Gualaxo do Sul, one at rio José Pedro, one at rio Piranga, three at rio Manhuaçu, one at rio Suaçuí Pequeno and five at rio Xopotó, all in the rio Doce Basin, Minas Gerais State, Brazil (Fig. 3).

#### Ecological notes.

*Neoplecostomus doceensis* is found in clear water rivers, varying from small to medium sized, with rocky outcrops forming small waterfalls and substrates of rocks and sand. The species is found at the bottom of the rivers among the rocks.

#### Etymology.

The specific name *doceensis* is a Latin noun meaning being located or having connection with the rio Doce basin. This hydrographic system is located in the southeastern region of Brazil and comprises a drainage area of 83,400 km², on the border of Minas Gerais and Espirito Santo states.

## Discussion

### Taxonomy

*Neoplecostomus doceensis* has a conspicuous series of enlarged papillae just posterior to the dentary teeth, which are larger than those on the remaining portions of the lower lip. The abdomen is covered with figlet shields of pentagonal, hexagonal or heptagonal shape. The canal bearing fig and the dorsal locking mechanism are absent, suggesting that this is a typical species of the genus *Neoplecostomus*, sensu Langeani (1990).

The main character used to distinguish the new species from its congeners is the enlarged fleshy folds between dentaries present in all specimens (Fig. 2a). Apparently, these folds can also be present in some large specimens of *Neoplecostomus selenae*, although it is poorly developed. Within *Neoplecostomus doceensis*, this character was observed in specimens of all sizes. However, it is more developed in mature males. Within *Neoplecostomus yapo*, we found variations of the folds between dentaries. In specimens of *Neoplecostomus yapo* of the rio Verde, municipality of Ponta Grossa (NUP 4300), this character is poorly developed, as in *Neoplecostomus selenae* and in specimens of the rio Atlântico, municipality of Mandaguaçu (NUP 4851), in which this character is absent. Several authors (e.g. Langeani 1990; Bizerril 1995; Zawadzki et al. 2008; Roxo et al. 2012c) have reported that the characters used to define the species of *Neoplecostomus* are influenced by both the sex and stage of ontogenetic development, which also occurs with the papillae between the dentary teeth.

The presence of two or three series of well-developed papillae anterior to the premaxillary teeth also distinguish the new species from its congeners (Fig. 2c). The presence of two or three series of conspicuous papillae just posterior to dentary teeth was previously discussed by Langeani (1990) and is used to diagnose the genus *Neoplecostomus*. Nevertheless, the papillae series anterior to premaxillary teeth have not previously been reported. Several species of *Neoplecostomus* such as *Neoplecostomus bandeirante*, *Neoplecostomus corumba* and *Neoplecostomus ribeirensis* have this character; however, it is best developed in *Neoplecostomus doceensis*. Apparently, this character is also influenced by sex and is enlarged in mature males.

### Biogeography and dispersal route

**Figure 4.**
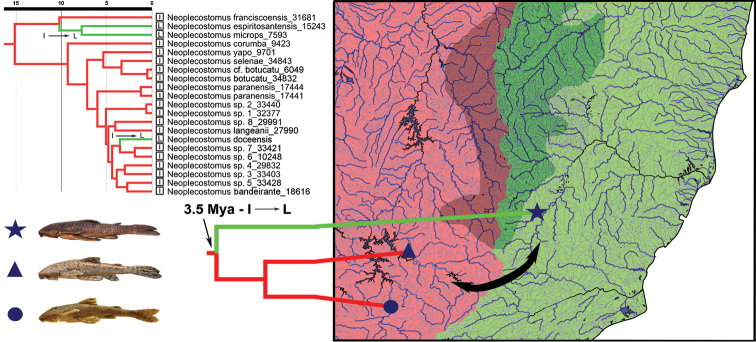
Distribution and time-calibrated phylogenetic tree of *Neoplecostomus* species, except *Neoplecostomus ribeirensis*, based on four mitochondrial (12S rRNA, 16SrRNA, COI, Cytb) and one nuclear marker (F-reticulon 4), modified from Figure 3 of Roxo et al. (2012a). The red coloration in the figure indicates the interior drainages (Upper Paraná, Iguassu, and São Francisco) and the green, the littoral drainages (Northeastern Mata Atlântica, Paraíba do Sul, Ribeira de Iguape, Southeastern Mata Atlântica and Fluminense). Based on our hypothesis, the ancestral lineage of *Neoplecostomus doceensis* dispersed from the upper rio Paraná to rio Doce drainages about 3.5 Mya (95% HPD: 1.6–5.5). See Table S1 in Roxo et al. (2012a) for all localities of undescribed species of *Neoplecostomus*.

Roxo et al. (2012a, 2012b), in a phylogenetic study of the Neoplecostominae species, suggested that *Neoplecostomus* originated within “interior running drainages” (i.e., drainages of the upper rio Paraná, rio Iguaçu, and rio São Francisco). An exception was found for *Neoplecostomus ribeirensis*, which appeared as a sister group to *Isbrueckerichthys* and originated in littoral drainages (i.e., Northeastern Mata Atlântica rivers, rio Paraíba do Sul, rio Ribeira de Iguape, Southeastern Mata Atlântica river, and Fluminense river). The new species, *Neoplecostomus doceensis* (cited as *Neoplecostomus* sp. 9 in Roxo et al. 2012a), is closely related to two undescribed species of *Neoplecostomus*, *Neoplecostomus* sp. 6 (from córrego do Sapateiro) and *Neoplecostomus* sp. 7 (from córrego Tamborete) both from streams in the rio Grande basin, an Atlantic coastal drainage. Roxo et al. (2012a) suggested that the ancestor of *Neoplecostomus doceensis* (cited as *Neoplecostomus* sp. 9) reached the rio Doce basin about 3.5 million years ago (95% HPD: 1.6–5.5) indicating a colonization route of the *Neoplecostomus doceensis* ancestral lineage from southern Serra do Espinhaço (Fig. 4), probably as a result of headwater capture processes between the upper rio Paraná and rio Doce basins.

Ribeiro (2006) suggested that the south-eastern region of Brazil has undergone intensive geological activity and that the activations of ancient faults could have resulted in headwater captures between adjacent drainages during several periods of its geological history. The eastern Brazilian coastal drainages have probably resulted in the capture of several adjacent rivers, including the headwaters of the Tietê, Grande, São Francisco and Doce rivers. A river capture event at this approximate time and place is also consistent with the General Area Cladogram of fish taxa from tropical South America (Albert and Carvalho 2011). This process is likely to have influenced the movement of ancestral fish throughout the adjacent drainages, similar to the geodispersal of the ancestor of *Neoplecostomus doceensis* from the upper rio Paraná drainages to the rio Doce drainages about 3.5 Mya (95% HPD: 1.6–5.5).

### Comparative material

*Neoplecostomus bandeirante*: holotype, MZUSP 110363, 109.9 mm SL, rio Paraitinguinha, rio Tietê basin, paratypes, LBP 3921, 2, 88.0–94.9 mm SL, rio Paraitinguinha, rio Tietê basin, LBP 2861, 8, 87.6–106.4 mm SL, rio Paraitinguinha, rio Tietê basin, NUP 6103, 1, 101.7 mm SL, rio Paraitinguinha, rio Tietê basin; *Neoplecostomus botucatu*: holotype, MZUSP 110364, 98.6 mm SL, córrego Águas de Madalena, rio Paranapanema basin, paratype, LBP 7525, 10, 80.3–102.2 mm SL, córrego Águas de Madalena, rio Paranapanema basin, LBP 8065, 12, 67.5–88.2 mm SL, córrego Águas de Madalena, rio Paranapanema basin, NUP 8016, 1, 69.8 mm SL, córrego Águas de Madalena, rio Paranapanema basin; *Neoplecostomus corumba*: holotype, DZSJRP 6713, 78.3 mm SL, córrego Gameleira, rio Paranaíba basin, paratypes, MZUSP 86208, 9, 45.7–77.6 mm SL, córrego Gameleira, rio Paranaíba basin; *Neoplecostomus espiritosantensis*: holotype, MZUSP 38573, 102.3 mm SL, rio Jucu, Coastal Drainage, LBP 2551, 2, 81.9–85.4 mm SL, rio Jucu, Coastal Drainage; *Neoplecostomus franciscoensis*: holotype, MZUSP 38577, 68.4 mm SL, affluent córrego Mutuca, rio São Francisco basin, LBP 6489, 50, 42.8–55.9 mm SL, rio das Velhas, rio São Francisco basin, MZUSP 107361, 7, 54.3-107.8 mm SL, rio Paraopeba, rio São Francisco basin; *Neoplecostomus langeanii*: holotype, MZUSP 110365, 85.5 mm SL, rio São Domingos, rio Grande basin, paratype, LBP 5931, 11, 48.4–69.6 mm SL, rio São Domingos, rio Grande basin, LBP 5947, 8, 56.6–73.5 mm SL, rio São Domingos, rio Grande basin; *Neoplecostomus microps*: LBP 6350, 4, 51.0–58.9 mm SL, rio Ribeirão Grande, rio Paraíba do Sul basin, LBP 8045, 31, 43.8–71.4 mm SL, ribeirão Piquete, rio Paraíba do Sul basin, LBP 8370, 17, 39.5–81.2 mm SL, rio Pomba, rio Paraíba do Sul basin; *Neoplecostomus paranensis*: holotype, MZUSP 38572, 71.4 mm SL, rio Cubatão, rio Grande basin, LBP 2732, 1, 70.5 mm SL, córrego Mocoquinha, rio Grande basin, MZUSP 10213, 2, 39.4–41.5 mm SL, rio Carandaí, rio Grande basin, MZUSP 35397, 1, 38.4 mm SL, rio São João, rio Grande basin, MZUSP 36583, 2, 52.0–62.4 mm SL, rio Cubatão, rio Grande basin, MZUSP 36625, 1, 56 mm SL, rio São Bartolomeu, rio Grande basin, MZUSP 38822, 1, 92.8 mm SL, rio Cubatão, rio Grande basin, MZUSP 38823, 1, 87.7 mm SL, rio Cubatão, rio Grande basin, MZUSP, 38824, 1, 68.1 mm SL, rio Cubatão, rio Grande basin; *Neoplecostomus ribeirensis*: LBP 7384, 16, 37.7–79.2 mm SL, rio Água Doce, rio Ribeira de Iguape basin; *Neoplecostomus selenae*: holotype, MZUSP 51889, 101.7 mm SL, ribeirão das Batéias, rio Paranapanema basin, paratype, DZSJRP 7449, 4, 56.5–95.8 mm SL, ribeirão das Batéias, rio Paranapanema basin, MZUSP 52589, 4, 42.8–64.9 mm SL, ribeirão das Batéias, rio Paranapanema basin, NUP 3572, 5, 48.0–84.8 mm SL, ribeirão das Batéias, rio Paranapanema basin; *Neoplecostomus yapo*: holotype, DZSJRP 6714, 97.4 mm SL, riacho Fortaleza, rio Paranapanema basin, paratype, MZUSP 86211, 7, 63.8–105.2 mm SL, affluent of rio Yapó, rio Paranapanema basin, NUP 2609, 15, 48.4–109.6 mm SL, riacho Fortaleza, rio Paranapanema basin, NUP 4300, 5, 73.5–89.1 mm SL, rio Verde, rio Paranapanema basin, NUP 4851, 11, 39.2–73.8 mm SL, rio Atlântico, rio Paranapanema basin.

## Supplementary Material

XML Treatment for Neoplecostomus doceensis
